# Human serum metabolic profiles are age dependent

**DOI:** 10.1111/j.1474-9726.2012.00865.x

**Published:** 2012-12

**Authors:** Zhonghao Yu, Guangju Zhai, Paula Singmann, Ying He, Tao Xu, Cornelia Prehn, Werner Römisch-Margl, Eva Lattka, Christian Gieger, Nicole Soranzo, Joachim Heinrich, Marie Standl, Elisabeth Thiering, Kirstin Mittelstraß, Heinz-Erich Wichmann, Annette Peters, Karsten Suhre, Yixue Li, Jerzy Adamski, Tim D Spector, Thomas Illig, Rui Wang-Sattler

**Affiliations:** 1 Research Unit of Molecular Epidemiology, Helmholtz Zentrum München85764 Neuherberg, Germany; 2 Department of Twin Research and Genetic Epidemiology, King’s College LondonLondon, UK; 3 Discipline of Genetics, Faculty of Medicine, Memorial University of NewfoundlandSt John’s, NL, Canada; 4 Shanghai Center for Bioinformation Technology200235 Shanghai, China; 5 Key Lab of Systems Biology, Shanghai Institutes for Biological Sciences, Chinese Academy of Sciences200031 Shanghai, China; 6 Genome Analysis Center, Institute of Experimental Genetics, Helmholtz Zentrum München85764 Neuherberg, Germany; 7 Institute of Bioinformatics and Systems Biology, Helmholtz Zentrum München85764 Neuherberg, Germany; 8 Institute of Genetic Epidemiology, Helmholtz Zentrum München85764 Neuherberg, Germany; 9 Wellcome Trust Sanger Institute Genome CampusHinxton, UK; 10 Institute of Epidemiology I, Helmholtz Zentrum München85764 Neuherberg, Germany; 11 Institute of Medical Informatics, Biometry and Epidemiology, Chair of Epidemiology, Ludwig-Maximilians-UniversitätMunich, Germany; 12 Klinikum GrosshadernMunich, Germany; 13 Institute of Epidemiology II, Helmholtz Zentrum München85764 Neuherberg, Germany; 14 Department of Environmental Health, Harvard School of Public Health Adjunct Associate Professor of Environmental EpidemiologyBoston, MA, USA; 15 Faculty of Biology, Ludwig-Maximilians-Universität82152 Planegg-Martinsried, Germany; 16 Department of Physiology and Biophysics, Weill Cornell Medical College in Qatar24144 Education City–Qatar Foundation, Doha, Qatar; 17 Institute of Experimental Genetics, Life and Food Science Center Weihenstephan, Technische Universität München85354 Freising-Weihenstephan, Germany; 18 Hannover Unified Biobank, Hannover Medical School30625 Hannover, Germany

**Keywords:** age, aging, epidemiology, metabolomics, population-based study

## Abstract

Understanding the complexity of aging is of utmost importance. This can now be addressed by the novel and powerful approach of metabolomics. However, to date, only a few metabolic studies based on large samples are available. Here, we provide novel and specific information on age-related metabolite concentration changes in human homeostasis. We report results from two population-based studies: the KORA F4 study from Germany as a discovery cohort, with 1038 female and 1124 male participants (32–81 years), and the TwinsUK study as replication, with 724 female participants. Targeted metabolomics of fasting serum samples quantified 131 metabolites by FIA-MS/MS. Among these, 71/34 metabolites were significantly associated with age in women/men (BMI adjusted). We further identified a set of 13 independent metabolites in women (with *P* values ranging from 4.6 × 10^−04^ to 7.8 × 10^−42^, α_corr_ = 0.004). Eleven of these 13 metabolites were replicated in the TwinsUK study, including seven metabolite concentrations that increased with age (C0, C10:1, C12:1, C18:1, SM C16:1, SM C18:1, and PC aa C28:1), while histidine decreased. These results indicate that metabolic profiles are age dependent and might reflect different aging processes, such as incomplete mitochondrial fatty acid oxidation. The use of metabolomics will increase our understanding of aging networks and may lead to discoveries that help enhance healthy aging.

## Introduction

Life expectancy in humans has dramatically increased throughout the world (Ferrucci *et al*., 2008). This process entails great challenges for world population in terms of health and economics, and complex social issues emerge, in particular for the future of healthcare systems, as aging is often accompanied by disabilities and diseases such as cardiovascular diseases, chronic lower respiratory tract disease, Alzheimer’s disease, chronic joint symptoms, arthritis, and diabetes (Butler *et al*., 2008; Wijsman *et al*., 2011). Thus, understanding the physiology of aging is of tremendous importance to allow populations to grow old, disease-free and with a good quality of life.

Aging is a very complex process because many transformations happen to the human organisms that affect all levels, from organ systems to cell organelles, and lead to a wide variety of altered functions. However, this process is incompletely understood. Genetic and environmental influences seem to be involved, but other approaches and insights are needed (Karasik *et al*., 2005; Piper & Bartke, 2008; Kerber *et al*., 2009; Deelen *et al*., 2011). Popular theories of aging include those implicating free radicals, accumulation of glycated proteins (AGEs), involvement of chronic low-grade inflammation, altered action of several hormones or chromosome telomere shortening (Szibor & Holtz, 2003; Franceschi *et al*., 2007; Yan *et al*., 2007; Jiang *et al*., 2008; Simm *et al*., 2008; Perheentupa & Huhtaniemi, 2009). Nonetheless, the largest volume of knowledge stems from non-human studies. Based on genetic studies in animal models, there are several complex pathways known to be involved in aging mechanisms that also are clearly linked to metabolism (Toth & Tchernof, 2000; Dennis *et al*., 2009; Feltes *et al*., 2011; Partridge *et al*., 2011).

Metabolomics is a key technology of modern systems biology that focuses on obtaining an integral depiction of the current metabolic status of an organism, associated with physiological and pathophysiological processes (Psychogios *et al*., 2011). Numerous small molecules are measured that can be both endogenous and exogenous. These ideally represent the whole range of intermediate metabolic pathways and may serve as biomarkers, indicating distinct physiological and/or pathophysiological states of an organism (He *et al*., 2012). Metabolomics is therefore a valuable tool for investigating in a single approach all the various ways in which metabolism is influenced, and then linking these influences to the phenotypic outcome of interest, thus a very promising tool for capturing the complexity of the aging process.

Only a few aging studies with metabolomics analyses have been conducted in animal models (in rats, mice and dogs) (Williams *et al*., 2005; Wang *et al*., 2007; Nevedomskaya *et al*., 2010). The very few published results on aging in adult humans have been small. A study of 269 individuals (both men and women) analyzed the human plasma metabolome with age using NMR and thus obtaining a very different set of metabolites to the current study (Lawton *et al*., 2008; Orešič, 2009). Nikkilä and colleagues performed a metabolomics study on early childhood, following 59 children from birth to an age of 4 years, and identified previously unknown metabolic changes with age (Nikkila *et al*., 2008).

The objectives of the current study were to characterize the metabolic profile of a large group of subjects with a wide age range (32–81 years) and identify metabolites relates to chronological age independent of BMI. Such metabolites may subsequently be investigated in future studies to establish whether they associate with health conditions and aging mechanisms.

## Results

### Study population

Individuals without metabolic diseases (e.g. hypertension, type 2 diabetes and obesity) were used in both studies of the Cooperative Health Research in the Region of Augsburg () (Holle *et al*., 2005; Wichmann *et al*., 2005) and the UK Adult Twin Registry (TwinsUK) (Spector & Williams, 2006; Moayyeri *et al*., 2012). KORA F4 sample involved 1038 women and 1124 men, aged 32–81 years (Table 1). Women and men of the KORA participants were about the same average age (54 years old) and had comparable mean BMIs (around 26 kg m^−2^). The TwinsUK contained 742 women, aged 19–82 years, with a mean age of 58 years and BMI of about 26 kg m^−2^. Comparing the discovery KORA women with the replication TwinsUK individuals, KORA women are about 4 years younger and have a slightly higher BMI (Table 1).

**Table 1:** Population characteristics of KORA F4 and Twins UK

	KORA F4		TwinsUK
		
	Males	Females	Females
*N*	1124	1038	742
Age (years)†	53.6 ± 12.5	54.1 ± 13.1	57.7 ± 10.6
BMI (kg m^−^²)†	25.9 ± 3.9	27.1 ± 3.2	25.6 ± 3.7

† Values of age and BMI are shown mean ± standard deviation (SD).

Serum concentrations of 163 metabolites were measured in all fasting participants; the 163 metabolites and their characteristics are summarized in Table S1 (Supporting information).

### Discovery of age-associated metabolites: general analyses procedure

Owing to prior results from KORA F4, which showed strong metabolic differences between women and men (Mittelstrass *et al*., 2011), we conducted strictly sex-separated analyses. Furthermore, we found that the BMI was significantly correlated with both age (Pearson’s *r* = 0.26, *P* = 2.2 × 10^−16^) and metabolite concentrations (Table S1) in KORA F4. To investigate the ‘true’ age-related metabolites, BMI was adjusted in subsequent analyses, and residuals of metabolite concentrations from linear regression against BMI were used in the graphs. We first plotted heat maps of mean residuals for 131 metabolites for each year of age as an explorative tool. Second, we used linear regression models for each metabolite at individual level to identify age-related metabolites. Moreover, as some of these metabolites correlated with each other, especially for the same classes (Fig. S1), we employed two additional statistical methods, the nonparametric random forest and the parametric stepwise selection methods, to identify unique and independent biomarker candidates. Finally, the set of age-related metabolites were replicated in the TwinsUK study. Smoother plots of this subset of metabolites were displayed to characterize age-associated changes regarding metabolite concentrations (Fig. S3–S5).

### Heat map of KORA F4 revealed association between age and metabolite

**Fig. 1:**
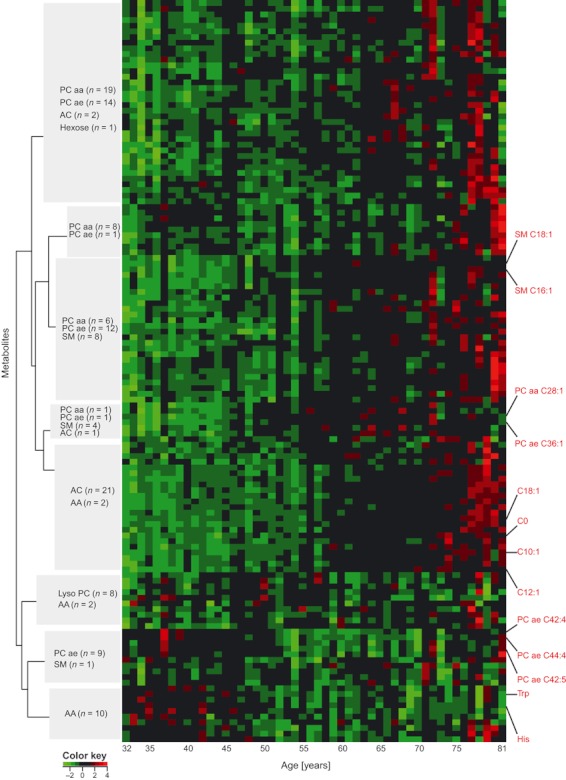
Heat map of the fold standard deviation changes between ages, and clustering of these changes, over all ages in 1038 women from KORA F4. The heat map shows changes of x-fold standard deviation from the overall mean concentration for each age year in a color-coded way. Green squares represent a decrease, and red squares an increase. Gray boxes represent groups of metabolites with similar changes with number of metabolites in parentheses. Metabolite names in red indicate our set of 13 metabolites. AA, amino acid; AC, acylcarnitines; PC aa, phosphatidylcholinediacyl; PC ae, phosphatidylcholine acyl-alkyl; and lyso PC a, lysophosphatidylcholine acyl.

We display heat maps of normalized mean metabolite residuals for each year of age in both women and men. The resulting heat map for women displayed a clear increase in metabolite concentrations with respect to age in KORA F4, in particular with most acylcarnitines (ACs) and diacyl phosphatidylcholines (PC aa), while others showed gradual decreases with age, for example, for most amino acids (AAs) (Fig. 1). A similar trend for most ACs and AAs were also observed in the heat map for men; however, most of the Lyso PCs decreased with age in men. In general, the results of heat maps of women and men showed different patterns of changes and clusters (the heat map result of men is shown in Fig. S2).

### Identification of a metabolite set for age

We performed multiple linear regression analysis between metabolite concentrations and age with BMI as covariate. In the discovery cohort, we found 71 metabolite concentrations in women and 34 metabolites in men that were significantly associated with age (*P* values < 3.8 × 10^−4^). As some of these metabolites are expected to correlate with each other, we employed two additional statistical methods (the nonparametric random forest and the parametric stepwise selection, see below) to identify independent biomarker candidates. Of the 71/34 metabolites, 13/12 were found to contain independent information in women and men, respectively (mean concentrations, beta and *P* values for women are shown in Table 2, and for men in Table S2) (Supporting information). Five metabolites (C12:1, C18:1, His, Trp, and PC ae C36:1) were found both in women and men.

**Table 2:** Potential biomarkers for aging in women from KORA F4 and TwinsUK

	Discovery sample			Replication sample				
				
	KORA F4 females			TwinsUK females			Meta-analysis	
			
Marker	Mean† ± SD	β‡ (SE)	*P* value§	Mean ± SD	β (SE)	*P* value§	β (SE)	*P* value§
C0	32.93 ± 6.79	0.45 (0.05)	2.77E−19	38.29 ± 9.38	0.26 (0.04)	5.30E−10¶	0.33 (0.03)	1.04E−26
C10:1	0.15 ± 0.05	79.67 (6.69)	7.96E−31	0.19 ± 0.06	24.15 (6.38)	7.53E−05¶	50.59 (4.62)	6.08E−28
C12:1	0.14 ± 0.04	105.20 (8.06)	2.93E−36	0.17 ± 0.05	28.64 (8.60)	1.00E−03¶	69.4 (5.88)	3.87E−32
C18:1	0.12 ± 0.03	118.02 (10.86)	3.65E−26	0.19 ± 0.05	33.09 (7.51)	5.40E−06¶	60.57 (6.18)	1.07E−22
His	97.41 ± 13.67	−0.18 (0.02)	3.15E−13	98.22 ± 28.93	−0.10 (0.02)	2.90E−06¶	−0.14 (0.01)	4.18E−23
Trp	80.2 ± 8.85	−0.24 (0.04)	1.29E−10	86.66 ± 16.63	−0.035 (0.03)	0.18	−0.11 (0.02)	5.81E−06
PC aa C28:1	3.56 ± 0.89	4.65 (0.36)	2.10E−35	4.17 ± 1.27	1.54 (0.33)	2.10E−06¶	2.96 (0.24)	4.59E−34
PC ae C36:1	8.94 ± 2.05	2.15 (0.15)	7.75E−42	12.14 ± 5.23	−0.31 (0.16)	2.00E−03	1 (0.11)	6.75E−20
PC ae C42:4	1.08 ± 0.25	−6.44 (1.39)	4.25E−06	1.18 ± 0.46	−5.84 (1.13)	1.20E−07¶	−6.08 (0.88)	4.13E−12
PC ae C42:5	2.49 ± 0.50	−2.42 (0.69)	4.57E−04	2.71 ± 0.98	−2.57 (0.52)	3.20E−07¶	−2.52 (0.42)	1.38E−09
PC ae C44:4	0.46 ± 0.11	−15.23 (3.05)	6.76E−07	0.51 ± 0.18	−10.56 (2.52)	1.00E−05¶	−12.45 (1.94)	1.45E−10
SM C16:1	16.80 ± 2.97	1.27 (0.11)	4.89E−28	19.58 ± 4.79	0.38 (0.09)	1.00E−05¶	0.74 (0.07)	3.74E−26
SM C18:1	11.96 ± 2.56	1.32 (0.13)	3.36E−22	12.38 ± 3.50	0.42 (0.12)	1.00E−03¶	0.33 (0.03)	1.04E−26

† Mean concentration in μm from serum.

‡ ß estimate represents changes per year of age, adjusted for BMI.

§ Corrected significance level of α_corr_ = 0.004 (correction for 13 tests according to Bonferroni method).

¶ Replication succeeded for these markers.

Smoother plots were drawn to characterize the trends and courses of metabolite concentration changes with age in women (Fig. S3) and men (Fig. S4). The smoothing method was ‘loess’, which is a locally weighted regression robust against a small fraction of outliers. As already stated for the heat map, some metabolite concentrations showed increasing, while some showed decreasing, trends with age, but smoother plots provided deeper insight. For example, the larger proportion of the identified metabolites showed linear associations with age for both women and men, with the exception of three metabolites in women (PC ae C42:4, PC ae 42:5, and PC ae C44:4) and one in men (Gln), which exhibited a decrease around age of 51.

### Replication of the metabolite set in the TwinsUK

For replication purpose, we used an independent sample of 742 women subjects derived from the TwinsUK cohort, for whom fasting serum metabolomics data were available. The metabolites showed comparable mean concentrations in both studies. Among the 13 age-related metabolites found in the KORA study, 11 metabolites were well replicated in the TwinsUK sample, with *P* < 0.004 for the significance level after adjustment for multiple testing with the Bonferroni correction method (Table 2), and the effect direction was the same as in the discovery sample (except for one metabolite, PC ae C36:1), with borderline significance in the TwinsUK sample (*P* = 2.00 × 10^−03^). We also drew smoother plots for the TwinsUK sample and obtained similar curve shapes (with the exception of PC ae C36:1) (Fig. S5).

Furthermore, we analyzed both study samples in a meta-analysis, which virtually gave the same results as in the KORA female sample alone. All 13 metabolites of the marker set were significant, with *P* values ranging from 5.18 × 10^−6^ to 3.87 × 10^−32^.

## Discussion

We found strong associations between age and the human metabolome and identified 11 metabolites that associated with age in women in both discovery and replication cohort. Our finding of 12 metabolites associated with age in men was not yet tested for confirmation.

The extent of sexual dimorphism in the metabolome has recently been shown (Mittelstrass *et al*., 2011), stressing the need for sex stratification. Even accounting for the lack of a replication sample, the results in men are less impressive for unclear reasons. Lifestyle and different life experiences probably play prominent roles in this. Nonetheless, five metabolites could be identified in both men and women in the discovery cohort, so that these may be considered to be metabolites associated with aging in the population as a whole.

We explored biological alterations that might have led to the observed changes in metabolite concentrations and could link our observations to common aging theories. As we had excluded participants with major metabolic diseases, we assume that our findings may be representative for the general aging population.

Phospholipids are major components of cell membranes. Changes in the cellular membrane that include a G protein coupled receptor may relate to aging, which affects at least G protein activity, cell morphology, and cell homeostasis (Naru *et al*., 2008).A common feature of cellular senescence is an increased cell surface. Naru *et al*. (2008) found that senescent cells had a higher uptake of PC species with long-chain fatty acid residues. Additionally, the number of special lipid rafts, termed caveolae, increased with senescence. Presumably, PC species were integrated into caveolae, which contain caveolin-1 as a crucial component capable of cell cycle suppression at the G0/G1 phase. Thus, altered consumption of phospholipids because of specific membranaceous demands could be associated with senescence, possibly involving the scavenger receptor SR-BI as the major mediator of selective phospholipid (PC, SM, and PE) uptake from particles as HDL and LDL (Engelmann & Wiedmann, 2010),

Sphingomyelins are further important components of cell membranes, especially neuronal cells, as they influence membrane fluidity and can promote signal transduction. While both Ichi *et al*. (2009) and Corre *et al*. (2010) reported the connection between oxidative stress and sphingomyelin metabolism, in their studies, oxidative stress was shown to accelerate degradation of sphingomyelins to ceramide, which would be inconsistent with our observation that elderly subjects had elevated SM levels. However, another study identified cells that had adapted to chronic oxidative stress by altering sphingomyelin metabolism and making major changes in membrane composition, leading to stabilization (Clement *et al*., 2009). The observed SM level elevations might indicate that aging humans have effective mechanisms to protect cells from oxidative stress, accompanied by changes in SM metabolism and incorporation of SM into cell membranes.

Elevated serum levels of acylcarnitines could be due to different underlying causes. AC species are found as a consequence of incompletely oxidized fatty acids because of an excess of beta oxidation capacity and related pathways (i.e. higher rate of substrate use than energy demand, with accumulated acyl-CoA converted to AC that then exits cells and tissues), but also as a consequence of oxidative stress (Noland *et al*., 2009). Acylcarnitines in turn show up in the blood. A recent rodent study suggested increased AC levels in blood could be healthy (Noland *et al*., 2009). In the study, Wistar rats maintained on a high-fat diet exhibited diminished carnitine and increased AC levels in skeletal muscle cells because of perturbations in mitochondrial fuel utilization, for example, they had incomplete fatty acid oxidation. Supplemented carnitine led to AC efflux, which in turn showed up in blood accompanied by improved metabolism and glucose tolerance. Thus, the carnitine shuttle system is considered to be a prominent factor in maintaining mitochondrial performance and glucose homeostasis.

The observed higher levels of AC with advanced age might indicate that the aging processes counteract oxidative damage from the mitochondria via the carnitine-acylcarnitine shuttle.

The metabolic fate of the amino acid histidine has two possible routes. Its presence in blood at higher ages could indicate an advanced tissue demand. The first histidine-consuming pathway is its metabolism to the biogenic amine histamine by a decarboxylation step. Histamine is involved in local immune responses and can act as neurotransmitter. However, to our knowledge, an association between histidine or histamine and immunosenescence has not been reported so far. The second metabolic pathway that consumes histidine produces carnosine. This dipeptide from beta-alanine and L-histidine is found in virtually all tissues, in particular in skeletal muscle cells and different brain cells (Derave *et al*., 2010). Owing to its antioxidant characteristics, carnosine is considered to be a natural anti-aging substance capable of suppressing oxidative damage, glycation of proteins, and scavenging toxic age-related molecules (Hipkiss, 2010). For instance, carnosine was shown to capture lipoxidation products and prevent protein cross-linking (Zhu *et al*., 2009). Assuming that histidine levels are lower owing to its consumption by carnosine biosynthesis with advancing age, decreased histidine in our study might reflect a response to oxidative stress.

Aging is understood as a continuous and dynamic remodeling process of the human organism accompanied by numerous losses and gains on different levels, including intermediate metabolism and cell function (Barbieri *et al*., 2009). Our results of a large number of age-associated metabolites and the replicated set of 11 most representative ones might reflect these processes and allow us to draw a more integral picture of the aging organism. Overall, we observed age-dependent differences in PC, SM, AC, and AA levels that might be linked to altered cell membrane composition, mitochondrial metabolism, and counteracting oxidative stress.

While the present study was large in participant number and metabolically well characterized, it should be noted that metabolite profiles were related to chronological not physiological age and that only a cross-sectional study design was used. It is also not yet known whether the changes in the 11 confirmed metabolites represent neutral changes with age, changes causal to physiological aspects of aging or beneficial responses to damaging agents. Further research on the metabolic surrounding of the metabolite set could eventually lead to early determination of a person’s potential for healthy aging at beginning of the remodeling process.

In summary, we identified a set of 11 significantly associated and replicated markers for age in women using the German KORA and TwinsUK studies. Literature gives indications that these markers might be linked to aging processes such as oxidative stress, alterations in cell morphology, beta oxidation capacity, and vascular function. This study shows the power of metabolomics to better understand the phenotype of aging in the human population and to link this knowledge in functional studies to aging pathways.

## Experimental procedures

### Sample Source

KORA is a population-based research platform with subsequent follow-up studies in the fields of epidemiology, health economics, and healthcare research (Holle *et al*., 2005; Wichmann *et al*., 2005). It is based on interviews and medical and laboratory examinations, as well as biological samples. Four surveys were conducted with 18 079 participants who live in the city of Augsburg (Southern Germany) and 16 surrounding towns and villages. KORA S4 consists of representative samples from 4261 individuals who live in Augsburg, who were examined during 1999–2001. During the years 2006–2008, 3080 participants took part in a follow-up (KORA F4) survey of the one conducted 7 years ago. For all studies, we obtained written consent from participants and approval from the ethics committee of the Bavarian medical association.

To avoid the potential influences from type 2 diabetes, hypertension and obesity, we excluded a total of 918 subjects from KORA F4 for subsequent analyses, resulting in 2162 subjects aged 32–81 years. Among the excluded were 20 experimental failures, 18 nonfasting subjects, 332 type 2 diabetics, 80 subjects without fasting glucose or 2 h glucose measurement, 77 subjects with systolic blood pressure > 160 mmHg, and 153 subjects with BMI > 35 kg m^−^². Further removals followed during statistical analyses (*n* = 239, see statistical section below).

### Sampling

Blood was drawn into serum gel tubes in the morning between 8:00 and 10:30 am after a fasting period of at least 8 h. Tubes were gently inverted twice, followed by 30 min resting at room temperature to obtain complete coagulation. For serum collection, blood was centrifuged at 2750 ***g*** at 15 °C for 10 min. Serum was frozen at −80 °C until execution of metabolic analyses.

### Metabolite measurements

The targeted metabolomics approach was based on measurements with the Absolute*IDQ*™ p150 kit (BIOCRATES Life Sciences AG, Innsbruck, Austria), allowing simultaneous quantification of 163 metabolites. The method conforms with FDA-Guidelines ‘Guidance for Industry – Bioanalytical Method Validation (May 2001)’, which implies proof of reproducibility within a given error range. The assay procedures and nomenclature have been described previously in detail (Zhai *et al*., 2010; Mittelstrass *et al*., 2011; Römisch-Margl *et al*., 2011; Yu *et al*., 2011). Metabolite measurements were adjusted for batch effect as we have described previously (Mittelstrass *et al*., 2011).

To ensure data quality, each metabolite had to meet the three same criteria we used before (Mittelstrass *et al*., 2011): (i) average value of the coefficient of variance (CV) for the metabolite in the three QCs should be smaller than 25%; (ii) 90% of all measured sample concentrations for the metabolite should be above the limit of detection (LOD); and (iii) the correlation coefficient between two duplicate measurements of the metabolite in 144 re-measured samples should be above 0.5. In total, 131 metabolites passed the three quality controls, and the final metabolomics dataset contained the sum of hexoses (H1), 14 amino acids (AA), 24 acylcarnitines (AC), 13 sphingomyelins (SMs), 34 diacyl phosphatidylcholines (PC aa), 37 acyl-alkyl PCs (PC ae), and eight lyso PCs. Table S1 (Supporting information) summarizes the characteristics of all 163 metabolites measured in KORA F4.

## Statistics

### Removal of outliers

To detect outliers, concentrations obtained for the remaining 131 metabolites were first scaled to have a mean of zero and a standard deviation (SD) of one and projected onto the unit sphere, and Mahalanobis distances for each individual were then calculated using the Robust principal components algorithm (Filzmoser *et al*., 2008) and were calculated separately for men and women. For each group, the mean Mahalanobis distance plus three times variance were defined as the cut-off. About 239 individuals whose distances were greater than these cut offs were identified as outliers.

### Residuals of metabolite concentrations

To avoid the influence of BMI when plotting the concentration of metabolites, we used the residuals from the linear regression model rather than use the absolute concentration. Log-transformed metabolite concentrations were treated as dependent, and the BMI as an independent, variable in the linear regression model, and the residuals from the model were used as the residuals for each metabolite concentrations. Regressions were performed for women and men separately.

### Heat map

Correlations were calculated for each metabolite pairs, and values were displayed using heat map. For participants with the same age, mean values were calculated for residuals from linear regression against BMI for each metabolite. These values were then scaled to a mean of 0 and a standard deviation of 1, to display a heat map that showed the changes of the metabolite concentrations with the increase of age; the color change in the heat map represents the concentration deviation from the mean value.

### Linear regression analysis

Linear regression was applied to model the relationship between age and the concentration of each metabolite, with BMI used as covariate. Metabolite concentrations were log-transformed to achieve normality. Regressions were done for men and women separately. To handle false discovery rates from multiple comparisons, the cut point for significance was calculated according to the Bonferroni correction, at a level of 3.8 × 10^−4^ (for a total use of 131 metabolites at 5% level).

### Smoother plots

Smoother plots were drawn for each metabolite of the set of metabolites with the R function ‘qplot’ (package ‘ggplot2’) using the options geom = smooth, method = loess, and span = 0.5, producing smoother plots with locally weighted regression (*loess*) applying a smoothing span of 0.5, which results in medium smoothing. The method computes outlier robust locally weighted regression fitted values by fitting local polynomials, using weights and results in the (loess) curve as shown in our smoother plots. Further information about the method has been previously published (Cleveland & Devlin 1988). For better visualization, plots were truncated to observations between the first and 99th percentiles.

### Criteria for metabolite selection

Multivariate linear regression, random forest, and a stepwise selection of linear regression methods were applied: metabolites were chosen if they both were significant in linear regression for every single metabolite, adjusting for BMI and also for the top 30 most important variables in random forest method, in which both the 131 metabolites and BMI were severed as variables. The chosen metabolites with BMI as co-variable were further selected based on a stepwise selection of multi-variables linear regression according to the Akaike information criterion (AIC) value.

All calculations were done with R statistical platform, version 2.12 (http://www.r-project.org/).

### Replication

TwinsUK is a UK-wide twin registry sample of 11 000 adults, founded in 1993 with the aim to explore the genetic epidemiology of common adult diseases (Spector & Williams, 2006). The cohort has been tested to be generalizable to UK population singletons, with no population stratification for a wide variety of musculoskeletal, CVD, or metabolic traits (Andrew *et al*., 2001). Over 7000 twins have attended detailed clinical examinations, with a wide range of phenotypes, over the last 18 years. Blood samples were taken after at least 6 h fasting at each visit. Samples were immediately inverted three times, followed by 40 min resting at 4 °C, to obtain complete coagulation. Samples were then centrifuged for 10 min at 1439 ***g***. Serum was removed from the centrifuged tubes as the top, yellow, clear layer of liquid. Aliquot in 4 × 1.5 mL skirted microcentrifuge tubes was then stored in a –45 °C freezer until sampling. About 1237 twins were selected for the targeted metabolomic profiling for either osteoarthritis or genetic studies. Metabolites were measured using the same metabolomics platform (Biocrates metabolomic assay kit; BIOCRATES Life Sciences AG, Innsbruck, Austria) and following an identical protocol as for the KORA study, at the Genome Analysis Centre of the Helmholtz Centre Munich (Zhai *et al*., 2010). To replicate the KORA F4 study, data on the 13 age-related metabolites identified in the KORA F4 aging study were retrieved, and the association between age and these 13 serum metabolites were analyzed by robust regression modeling that takes into account twin relatedness.

Of the data on a total of 1237 individuals with metabolomic data available, 44 men were excluded. Following the KORA F4 study’s exclusion criteria, we further excluded 64 individuals with systolic blood pressure > 160 mmHg, 14 individuals with type 2 diabetes, 45 individuals with BMI > 35 kg m^−2^, and 328 individuals for whom no data for blood pressure, fasting serum glucose levels, or diabetes diagnosis data were available. A total of 742 female individuals were included in the final analysis.

### Meta-analysis

For the meta-analyses of KORA and TwinsUK women, a fixed effects model was used.

## Supporting Information

**Fig. S1** Correlation matrix for women in KORA F4.

**Fig. S2** Heat map of the fold standard deviation changes between ages, and clustering of these changes over all ages, in 1124 males from KORA F4.

**Fig. S3** Concentration trends of 13 potential biomarkers in women from KORA F4.

**Fig. S4** Concentration trends of 12 selected metabolites in males from KORA F4.

**Fig. S5** Concentration trends of the set of 13 metabolites in women from the TwinsUK study.

**Table S1** Characteristics of 163 metabolites in KORA F4.

**Table S2** Potential biomarkers for age in men from KORA F4.
