# Supplementary figures and images for: Human serum metabolic profiles are age dependent
Source: Aging Cell. 2012 Dec;11(6):960–7. doi: 10.1111/j.1474-9726.2012.00865.x (PMC3533791; doi:10.1111/j.1474-9726.2012.00865.x)

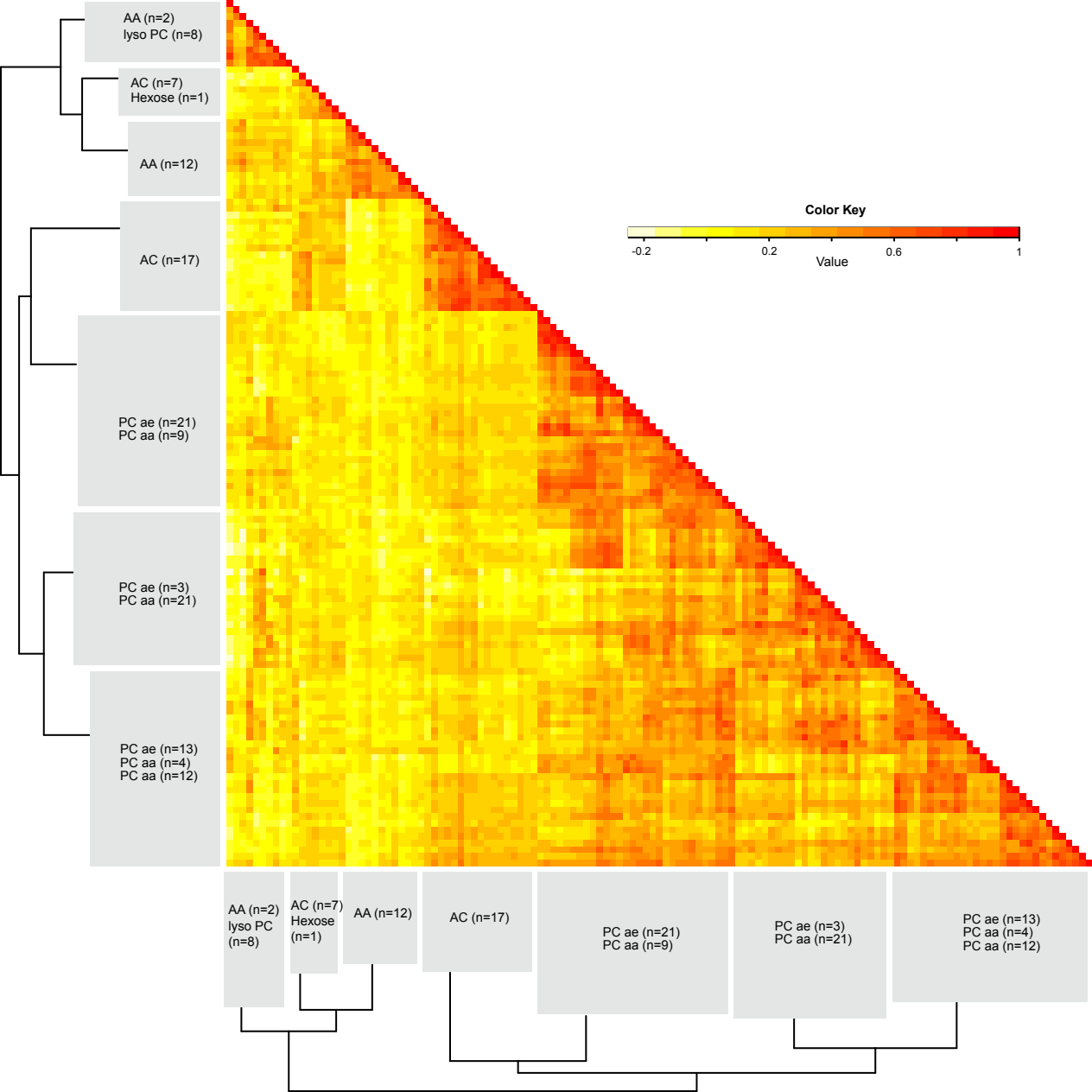

Supplement: Supplementary file 1 [file acel0011-0960-SD1.pdf]

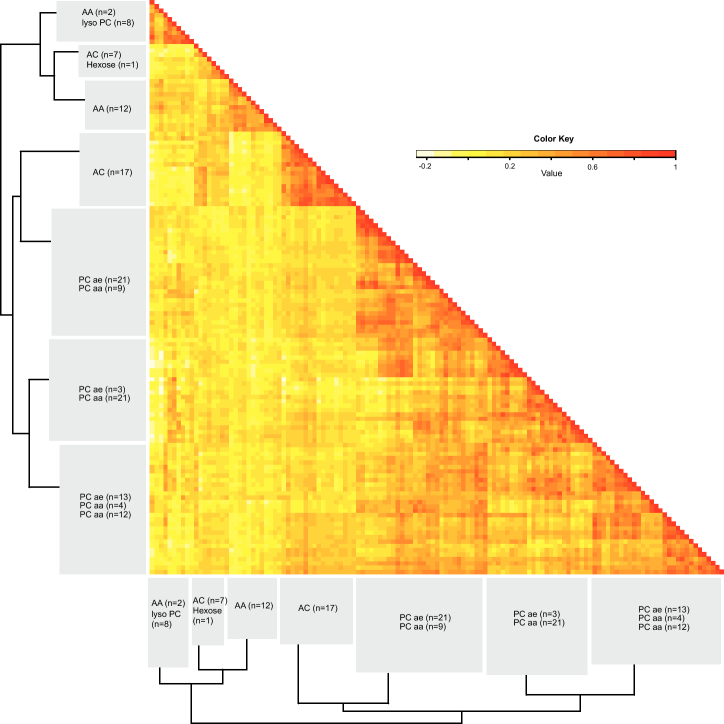

Supplement: Supplementary file 2 [file acel0011-0960-SD7.png]

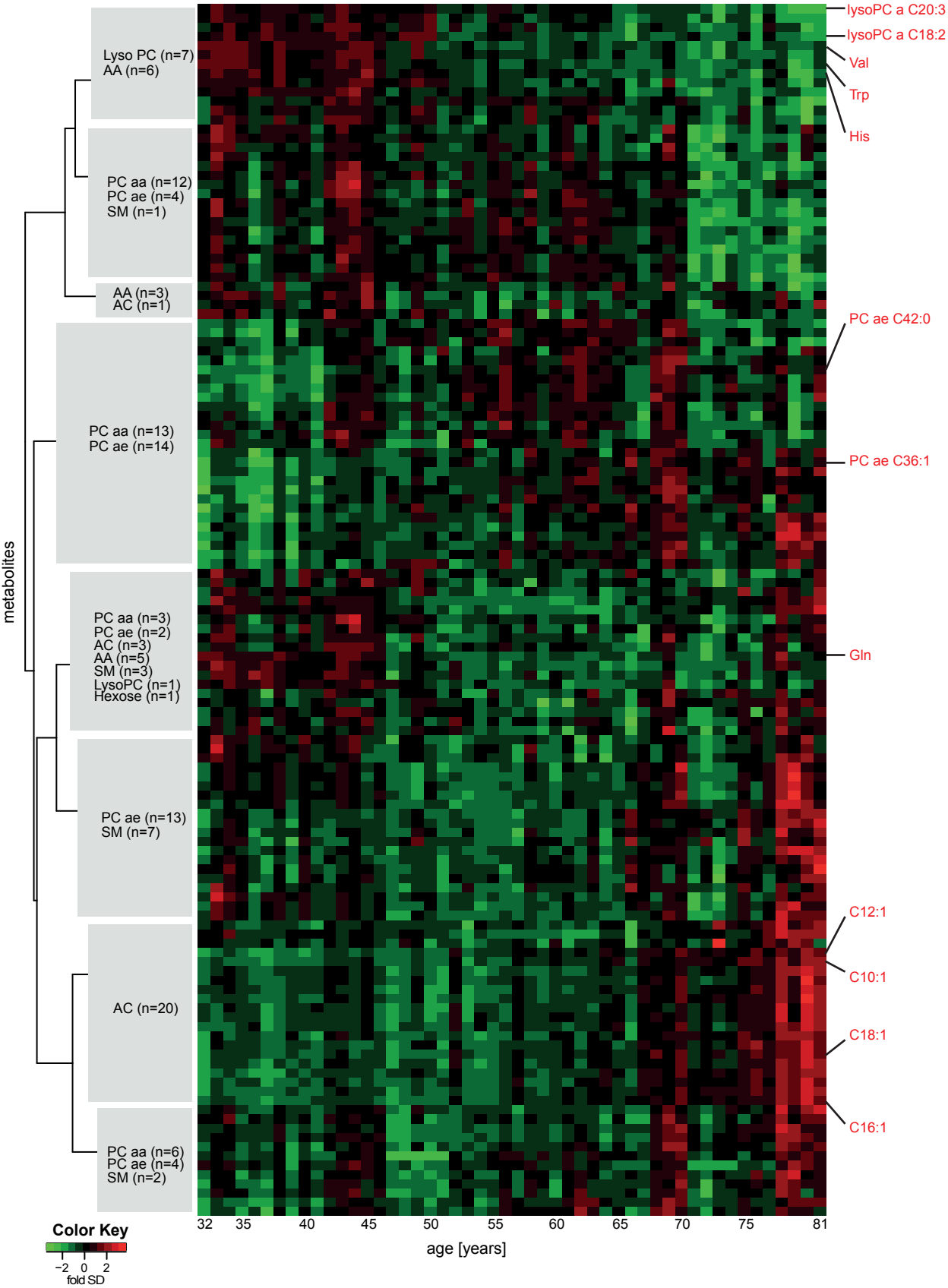

Supplement: Supplementary file 4 [file acel0011-0960-SD2.pdf]

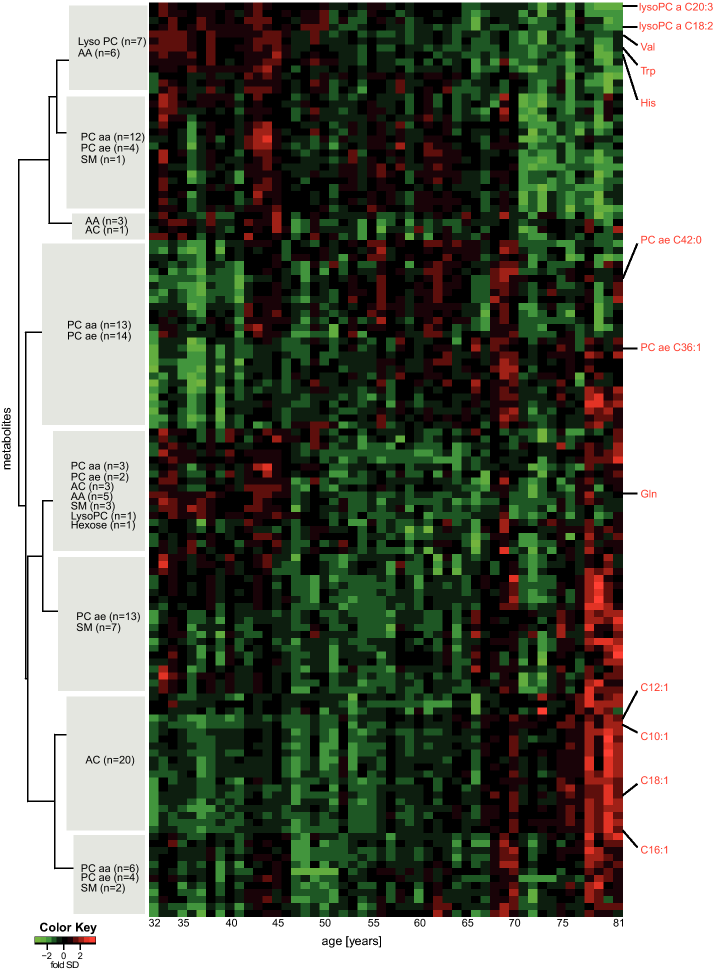

Supplement: Supplementary file 5 [file acel0011-0960-SD8.png]

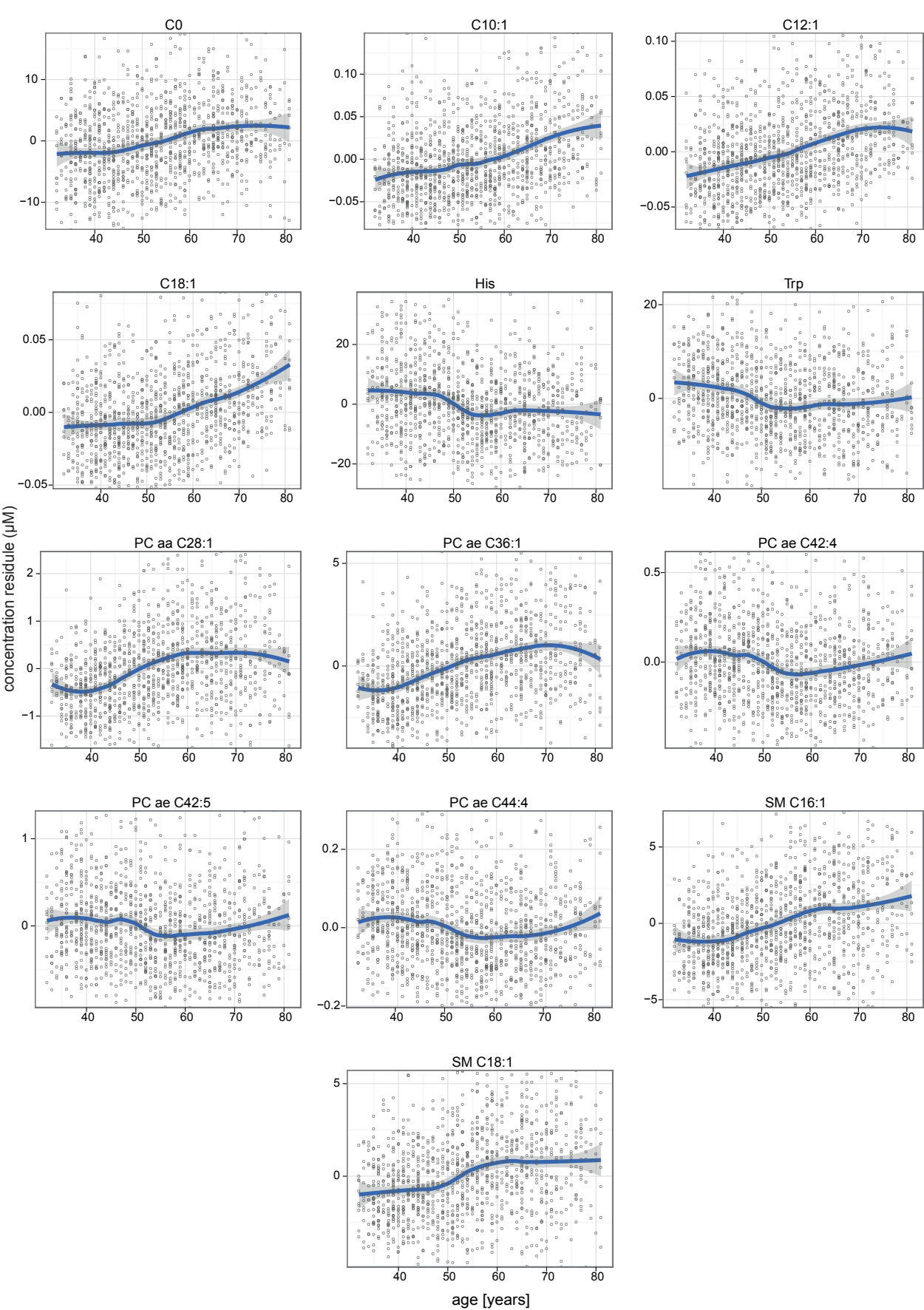

Supplement: Supplementary file 7 [file acel0011-0960-SD3.pdf]

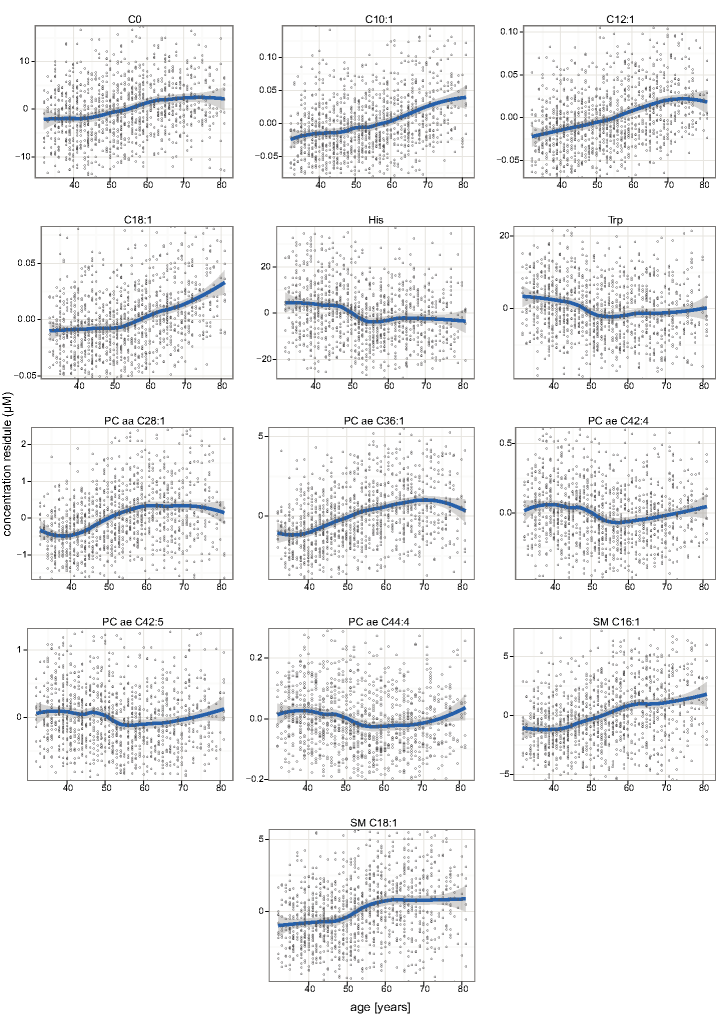

Supplement: Supplementary file 8 [file acel0011-0960-SD9.png]

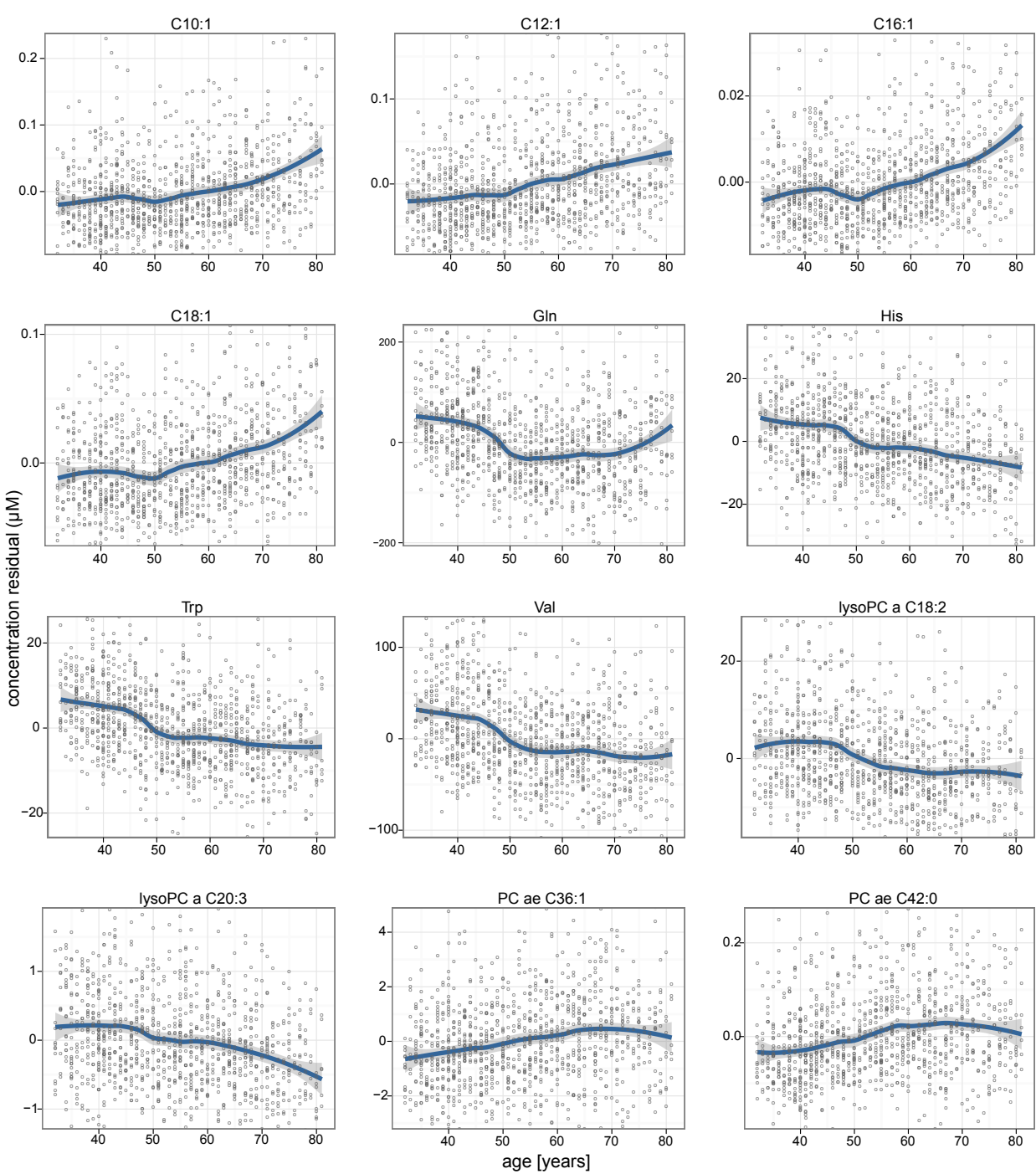

Supplement: Supplementary file 10 [file acel0011-0960-SD4.pdf]

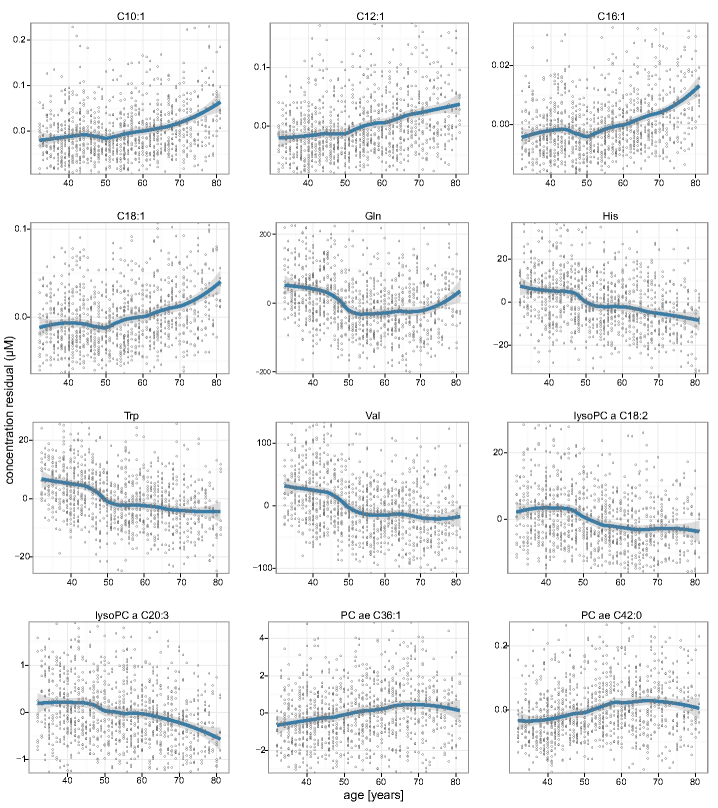

Supplement: Supplementary file 11 [file acel0011-0960-SD10.png]

C0

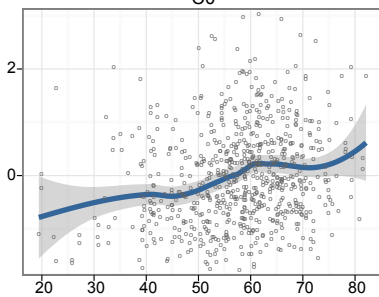

C10:1

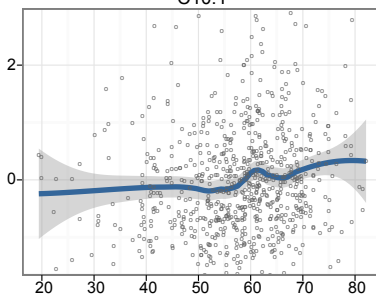

C12:1

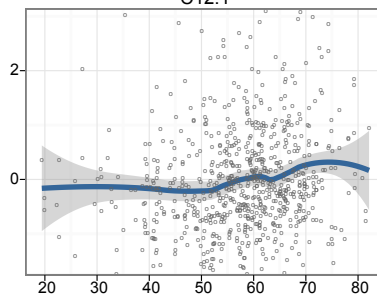

C18:1

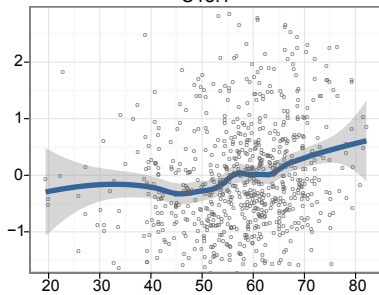

His

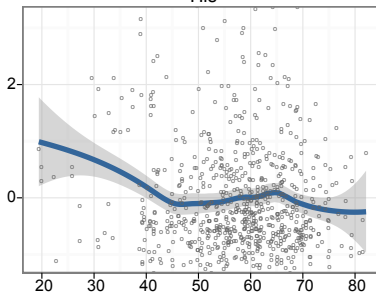

Trp

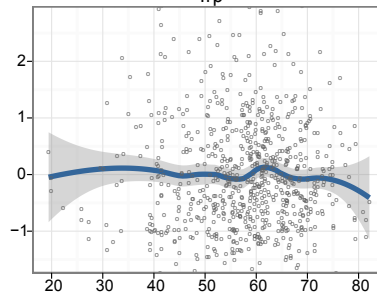

PC aa C28:1

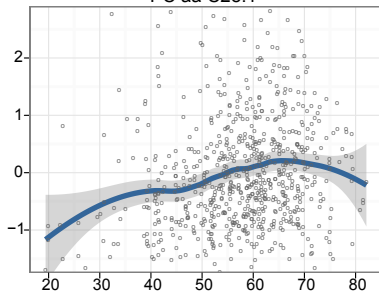

PC ae C36:1

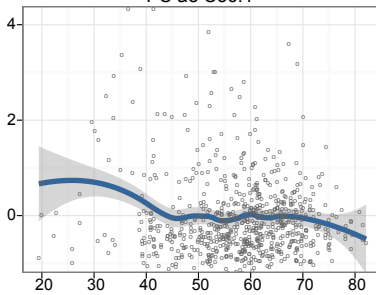

PC ae C42:4

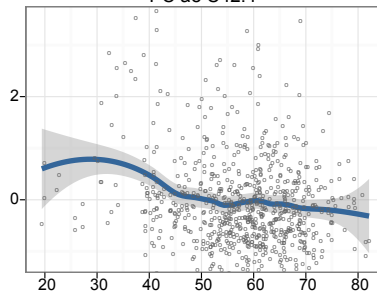

PC ae C42:5

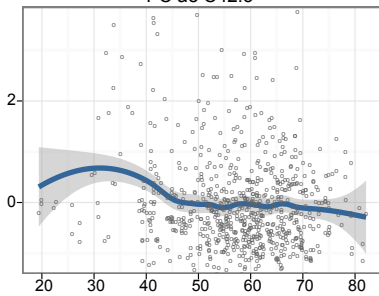

PC ae C44:4

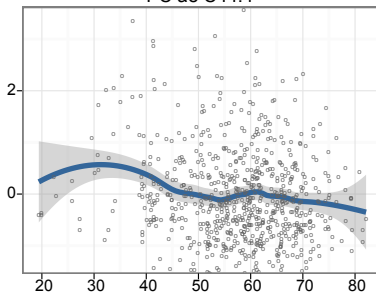

SM C16:1

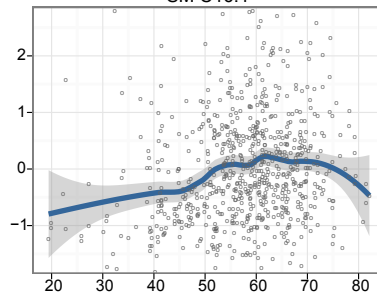

SM C18:1

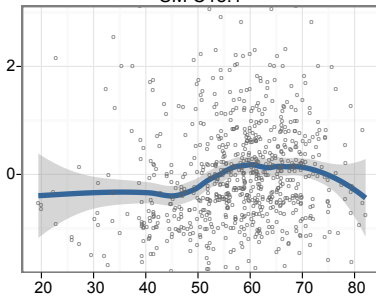

age [years]

Supplement: Supplementary file 13 [file acel0011-0960-SD5.pdf]

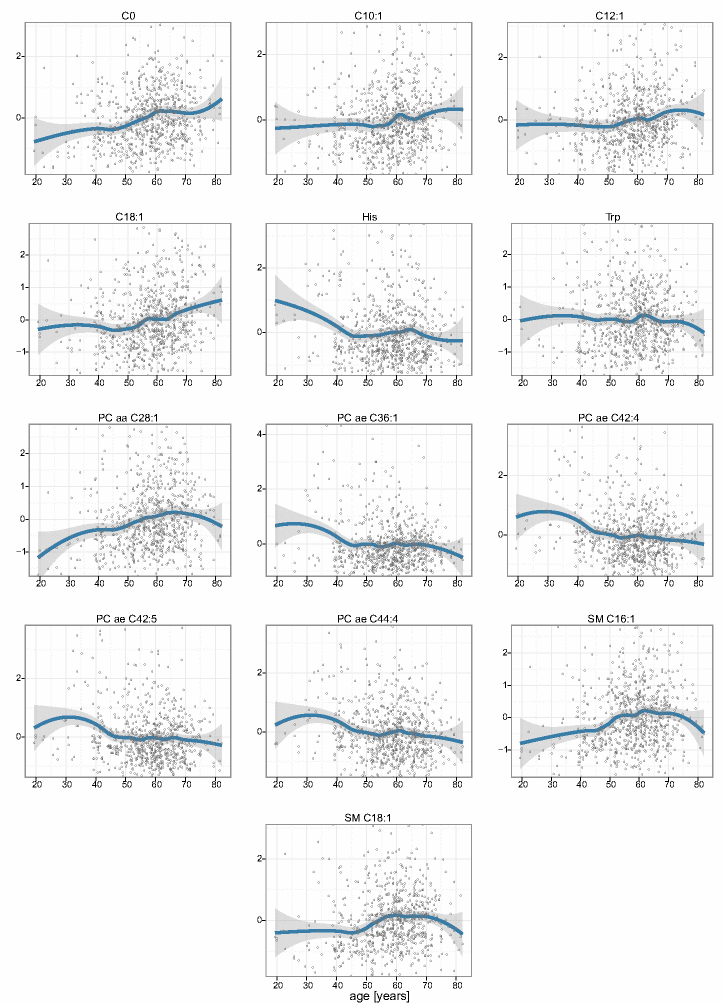

Supplement: Supplementary file 14 [file acel0011-0960-SD11.png]
