# Supplementary material for: Human serum metabolic profiles are age dependent
Source: Aging Cell. 2012 Dec;11(6):960–7. doi: 10.1111/j.1474-9726.2012.00865.x (PMC3533791; doi:10.1111/j.1474-9726.2012.00865.x)
Supplement: Supplementary file 16 [file acel0011-0960-SD6.doc]

**Table S1│ Characteristics of the 163 targeted metabolites**

| **Abbreviation** | **Biochemical name** | **r** | **Above LOD (%)** | **CV** | **Application** | **Mean concentration and standard deviation** | | **r between metabolite concentration and BMI** | |
| --- | --- | --- | --- | --- | --- | --- | --- | --- | --- |
|  |  |  |  |  |  | **Females** | **Males** | **Males** | **Females** |
| C0 | Carnitine | 0.88 | 100.00 | 6.7% | Used | 33.03 ± 6.83 | 38.18 ± 7.38 | 0.24 | 0.18 |
| C2 | Acetylcarnitine | 0.94 | 100.00 | 9.4% | Used | 7.81 ± 2.41 | 8.04 ± 2.78 | 0.11 | 0.08 |
| C3 | Propionylcarnitine | 0.86 | 100.00 | 8.0% | Used | 0.35 ± 0.1 | 0.43 ± 0.12 | 0.26 | 0.16 |
| C3‐OH | Hydroxypropionylcarnitine | 0.05 | 0.36 | 76.6% | Excluded |  |  |  |  |
| C3:1 | Propenonylcarnitine | -0.11 | 0.10 | 37.5% | Excluded |  |  |  |  |
| C4 | Butyrylcarnitine | 0.89 | 100.00 | 8.8% | Used | 0.21 ± 0.09 | 0.24 ± 0.12 | 0.15 | 0.03 |
| C4‐OH(C3‐DC) | Hydroxybutyrylcarnitine | 0.47 | 8.40 | 35.5% | Excluded |  |  |  |  |
| C4:1 | Butenylcarnitine | 0.04 | 5.65 | 34.7% | Excluded |  |  |  |  |
| C5 | Valerylcarnitine | 0.81 | 95.56 | 14.2% | Used | 0.1 ± 0.03 | 0.13 ± 0.04 | 0.16 | 0.15 |
| C5‐DC(C6‐OH) | Glutarylcarnitine (Hydroxyhexanoylcarnitine) | 0.15 | 27.06 | 21.0% | Excluded |  |  |  |  |
| C5‐M‐DC | Methylglutarylcarnitine | 0.18 | 0.95 | 42.9% | Excluded |  |  |  |  |
| C5‐OH (C3‐DC‐M) | Hydroxyvalerylcarnitine (Methylmalonylcarnitine) | 0.25 | 55.10 | 28.7% | Excluded |  |  |  |  |
| C5:1 | Tiglylcarnitine | 0.37 | 0.75 | 26.1% | Excluded |  |  |  |  |
| C5:1‐DC | Glutaconylcarnitine | 0.13 | 12.48 | 42.4% | Excluded |  |  |  |  |
| C6(C4:1‐DC) | Hexanoylcarnitine (Fumarylcarnitine) | 0.85 | 76.67 | 13.6% | Used | 0.07 ± 0.02 | 0.07 ± 0.03 | 0.14 | 0.12 |
| C6:1 | Hexenoylcarnitine | 0.07 | 0.33 | 32.4% | Excluded |  |  |  |  |
| C7‐DC | Pimelylcarnitine | 0.79 | 61.34 | 34.4% | Excluded |  |  |  |  |
| C8 | Octanoylcarnitine | 0.89 | 51.54 | 16.3% | Used | 0.2 ± 0.07 | 0.23 ± 0.11 | 0.04 | 0.03 |
| C8:1 | Octenoylcarnitine | 0.92 | 96.01 | 8.4% | Used | 0.08 ± 0.04 | 0.09 ± 0.05 | 0.24 | 0.11 |
| C9 | Nonaylcarnitine | 0.84 | 83.73 | 20.8% | Used | 0.05 ± 0.02 | 0.05 ± 0.03 | -0.01 | -0.12 |
| C10 | Decanoylcarnitine | 0.93 | 94.08 | 11.4% | Used | 0.33 ± 0.12 | 0.38 ± 0.2 | -0.03 | -0.01 |
| C10:1 | Decenoylcarnitine | 0.83 | 48.66 | 10.4% | Used | 0.15 ± 0.05 | 0.17 ± 0.06 | 0.10 | 0.01 |
| C10:2 | Decadienylcarnitine | 0.51 | 50.49 | 14.5% | Used | 0.04 ± 0.01 | 0.04 ± 0.01 | 0.16 | 0.01 |
| C12 | Dodecanoylcarnitine | 0.86 | 87.35 | 10.4% | Used | 0.12 ± 0.04 | 0.14 ± 0.05 | -0.06 | -0.06 |
| C12‐DC | Dodecanedioylcarnitine | 0.05 | 0.00 | 12.2% | Excluded |  |  |  |  |
| C12:1 | Dodecenoylcarnitine | 0.73 | 13.69 | 13.0% | Used | 0.14 ± 0.04 | 0.15 ± 0.05 | 0.03 | 0.02 |
| C14 | Tetradecanoylcarnitine | 0.54 | 51.67 | 12.6% | Used | 0.04 ± 0.01 | 0.05 ± 0.01 | 0.02 | -0.01 |
| C14:1 | Tetradecenoylcarnitine | 0.81 | 100.00 | 16.9% | Used | 0.14 ± 0.03 | 0.15 ± 0.04 | 0.03 | -0.01 |
| C14:1‐OH | Hydroxytetradecenoylcarnitine | 0.70 | 67.35 | 16.4% | Used | 0.01 ± 0 | 0.02 ± 0 | 0.05 | 0.01 |
| C14:2 | Tetradecadienylcarnitine | 0.87 | 98.82 | 11.6% | Used | 0.03 ± 0.01 | 0.03 ± 0.01 | 0.01 | -0.05 |
| C14:2‐OH | Hydroxytetradecadienylcarnitine | 0.27 | 38.04 | 17.4% | Excluded |  |  |  |  |
| C16 | Hexadecanoylcarnitine | 0.84 | 100.00 | 8.9% | Used | 0.11 ± 0.02 | 0.12 ± 0.03 | 0.15 | 0.09 |
| C16‐OH | Hydroxyhexadecanoylcarnitine | 0.20 | 3.33 | 24.1% | Excluded |  |  |  |  |
| C16:1 | Hexadecenoylcarnitine | 0.71 | 2.78 | 10.2% | Used | 0.04 ± 0.01 | 0.04 ± 0.01 | 0.10 | 0.07 |
| C16:1‐OH | Hydroxyhexadecenoylcarnitine | 0.38 | 2.25 | 17.5% | Excluded |  |  |  |  |
| C16:2 | Hexadecadienylcarnitine | 0.57 | 70.69 | 19.4% | Used | 0.01 ± 0 | 0.01 ± 0 | 0.08 | 0.02 |
| C16:2‐OH | Hydroxyhexadecadienylcarnitine | 0.32 | 4.67 | 16.6% | Excluded |  |  |  |  |
| C18 | Octadecanoylcarnitine | 0.69 | 99.80 | 13.7% | Used | 0.05 ± 0.01 | 0.05 ± 0.01 | 0.00 | -0.03 |
| C18:1 | Octadecenoylcarnitine | 0.87 | 98.33 | 10.2% | Used | 0.12 ± 0.03 | 0.13 ± 0.04 | 0.14 | 0.07 |
| C18:1‐OH | Hydroxyoctadecenoylcarnitine | 0.06 | 0.95 | 33.4% | Excluded |  |  |  |  |
| C18:2 | Octadecadienylcarnitine | 0.81 | 100.00 | 9.4% | Used | 0.04 ± 0.01 | 0.05 ± 0.01 | 0.11 | 0.01 |
| Arg | Arginine | 0.59 | 100.00 | 8.2% | Used | 114.62 ± 18.68 | 116.9 ± 19.23 | 0.07 | 0.04 |
| Gln | Glutamine | 0.62 | 100.00 | 9.9% | Used | 603.76 ± 89.22 | 636.48 ± 94.87 | -0.05 | -0.16 |
| Gly | Glycine | 0.89 | 100.00 | 7.9% | Used | 338.26 ± 93.83 | 289.7 ± 59.72 | -0.17 | -0.25 |
| His | Histidine | 0.69 | 100.00 | 8.3% | Used | 97.29 ± 13.72 | 100.83 ± 14.18 | -0.10 | -0.04 |
| Met | Methionine | 0.53 | 100.00 | 9.7% | Used | 29.9 ± 5.15 | 33.34 ± 5.63 | -0.01 | 0.04 |
| Orn | Ornithine | 0.75 | 100.00 | 9.4% | Used | 77.26 ± 18.36 | 83.85 ± 18.07 | 0.16 | 0.06 |
| Phe | Phenylalanine | 0.62 | 100.00 | 8.4% | Used | 58.99 ± 9.17 | 63.38 ± 10.09 | 0.18 | 0.18 |
| Pro | Proline | 0.89 | 100.00 | 7.4% | Used | 155.88 ± 48.32 | 190.99 ± 50.38 | 0.12 | 0.00 |
| Ser | Serine | 0.62 | 100.00 | 9.6% | Used | 133.06 ± 25.78 | 125.55 ± 22.24 | -0.13 | -0.10 |
| Thr | Threonine | 0.71 | 100.00 | 12.1% | Used | 105.74 ± 26.18 | 107.92 ± 22.51 | -0.10 | -0.05 |
| Trp | Tryptophan | 0.51 | 100.00 | 7.5% | Used | 80.23 ± 8.87 | 85.76 ± 10.23 | -0.01 | 0.01 |
| Tyr | Tyrosine | 0.66 | 100.00 | 8.6% | Used | 79.93 ± 16.7 | 87.41 ± 16.77 | 0.27 | 0.31 |
| Val | Valine | 0.69 | 100.00 | 19.6% | Used | 247.58 ± 43.66 | 293.31 ± 51.56 | 0.26 | 0.15 |
| xLeu | Leucine/Isoleucine | 0.74 | 100.00 | 8.2% | Used | 186.94 ± 31.88 | 234.26 ± 41.15 | 0.22 | 0.16 |
| PC aa C24:0 | Phosphatidylcholine diacyl C24:0 | 0.11 | 72.55 | 26.5% | Excluded |  |  |  |  |
| PC aa C26:0 | Phosphatidylcholine diacyl C26:0 | 0.09 | 11.54 | 32.9% | Excluded |  |  |  |  |
| PC aa C28:1 | Phosphatidylcholine diacyl C28:1 | 0.87 | 100.00 | 9.8% | Used | 3.58 ± 0.89 | 3.15 ± 0.72 | 0.17 | 0.04 |
| PC aa C30:0 | Phosphatidylcholine diacyl C30:0 | 0.89 | 100.00 | 7.8% | Used | 4.94 ± 1.48 | 4.43 ± 1.38 | -0.01 | 0.04 |
| PC aa C30:2 | Phosphatidylcholine diacyl C30:2 | 0.12 | 4.22 | 81.6% | Excluded |  |  |  |  |
| PC aa C32:0 | Phosphatidylcholine diacyl C32:0 | 0.83 | 100.00 | 7.1% | Used | 15.1 ± 3 | 14.72 ± 3.04 | 0.00 | 0.06 |
| PC aa C32:1 | Phosphatidylcholine diacyl C32:1 | 0.96 | 100.00 | 7.4% | Used | 21.76 ± 9.91 | 20.47 ± 11.71 | 0.04 | 0.15 |
| PC aa C32:2 | Phosphatidylcholine diacyl C32:2 | 0.91 | 99.93 | 11.1% | Used | 4.21 ± 1.6 | 3.53 ± 1.44 | 0.02 | 0.07 |
| PC aa C32:3 | Phosphatidylcholine diacyl C32:3 | 0.79 | 100.00 | 8.9% | Used | 0.52 ± 0.12 | 0.44 ± 0.09 | 0.15 | -0.02 |
| PC aa C34:1 | Phosphatidylcholine diacyl C34:1 | 0.83 | 100.00 | 7.2% | Used | 238.52 ± 51.07 | 237.49 ± 55.58 | -0.05 | 0.07 |
| PC aa C34:2 | Phosphatidylcholine diacyl C34:2 | 0.75 | 100.00 | 7.7% | Used | 393.26 ± 59.69 | 381.85 ± 64.88 | -0.05 | 0.05 |
| PC aa C34:3 | Phosphatidylcholine diacyl C34:3 | 0.91 | 100.00 | 8.6% | Used | 19.18 ± 5.09 | 16.69 ± 4.89 | -0.03 | 0.03 |
| PC aa C34:4 | Phosphatidylcholine diacyl C34:4 | 0.92 | 100.00 | 8.0% | Used | 2.37 ± 0.83 | 2.13 ± 0.73 | 0.09 | 0.11 |
| PC aa C36:0 | Phosphatidylcholine diacyl C36:0 | 0.74 | 100.00 | 17.4% | Used | 2.73 ± 0.75 | 2.67 ± 0.77 | -0.06 | -0.05 |
| PC aa C36:1 | Phosphatidylcholine diacyl C36:1 | 0.84 | 100.00 | 8.5% | Used | 53.98 ± 12.22 | 52.69 ± 13.64 | 0.06 | 0.09 |
| PC aa C36:2 | Phosphatidylcholine diacyl C36:2 | 0.80 | 100.00 | 6.7% | Used | 236.47 ± 39.51 | 226.5 ± 42.86 | 0.04 | 0.07 |
| PC aa C36:3 | Phosphatidylcholine diacyl C36:3 | 0.86 | 100.00 | 7.5% | Used | 152.43 ± 29.67 | 144.83 ± 29.9 | 0.06 | 0.12 |
| PC aa C36:4 | Phosphatidylcholine diacyl C36:4 | 0.87 | 100.00 | 7.8% | Used | 216.96 ± 48.45 | 216.32 ± 51.28 | 0.06 | 0.11 |
| PC aa C36:5 | Phosphatidylcholine diacyl C36:5 | 0.82 | 100.00 | 8.6% | Used | 28.55 ± 12.89 | 29.23 ± 14.04 | 0.08 | 0.01 |
| PC aa C36:6 | Phosphatidylcholine diacyl C36:6 | 0.89 | 100.00 | 11.1% | Used | 1.18 ± 0.43 | 1.06 ± 0.41 | 0.03 | 0.00 |
| PC aa C38:0 | Phosphatidylcholine diacyl C38:0 | 0.86 | 100.00 | 13.8% | Used | 3.38 ± 0.89 | 3.2 ± 0.84 | -0.02 | -0.06 |
| PC aa C38:1 | Phosphatidylcholine diacyl C38:1 | 0.34 | 99.84 | 18.1% | Excluded |  |  |  |  |
| PC aa C38:3 | Phosphatidylcholine diacyl C38:3 | 0.86 | 100.00 | 7.6% | Used | 54.06 ± 12.59 | 51.54 ± 12.76 | 0.35 | 0.30 |
| PC aa C38:4 | Phosphatidylcholine diacyl C38:4 | 0.88 | 100.00 | 7.3% | Used | 117.82 ± 28.03 | 116.33 ± 31 | 0.21 | 0.20 |
| PC aa C38:5 | Phosphatidylcholine diacyl C38:5 | 0.83 | 100.00 | 7.9% | Used | 62.11 ± 14.07 | 61.25 ± 15.12 | 0.06 | 0.04 |
| PC aa C38:6 | Phosphatidylcholine diacyl C38:6 | 0.93 | 100.00 | 8.1% | Used | 90.79 ± 25.93 | 87.98 ± 25.49 | 0.02 | 0.05 |
| PC aa C40:1 | Phosphatidylcholine diacyl C40:1 | 0.51 | 8.66 | 13.5% | Used | 0.48 ± 0.09 | 0.46 ± 0.09 | -0.06 | -0.10 |
| PC aa C40:2 | Phosphatidylcholine diacyl C40:2 | 0.51 | 100.00 | 11.7% | Used | 0.36 ± 0.09 | 0.35 ± 0.09 | -0.03 | -0.01 |
| PC aa C40:3 | Phosphatidylcholine diacyl C40:3 | 0.60 | 100.00 | 11.2% | Used | 0.66 ± 0.14 | 0.64 ± 0.15 | 0.00 | 0.01 |
| PC aa C40:4 | Phosphatidylcholine diacyl C40:4 | 0.86 | 100.00 | 7.6% | Used | 4.04 ± 1.05 | 4.11 ± 1.24 | 0.12 | 0.19 |
| PC aa C40:5 | Phosphatidylcholine diacyl C40:5 | 0.89 | 100.00 | 7.0% | Used | 11.2 ± 2.89 | 11.41 ± 3.26 | 0.14 | 0.18 |
| PC aa C40:6 | Phosphatidylcholine diacyl C40:6 | 0.93 | 100.00 | 7.1% | Used | 28.2 ± 9.06 | 27.7 ± 9.14 | 0.19 | 0.18 |
| PC aa C42:0 | Phosphatidylcholine diacyl C42:0 | 0.85 | 99.97 | 12.3% | Used | 0.64 ± 0.18 | 0.57 ± 0.18 | -0.16 | -0.21 |
| PC aa C42:1 | Phosphatidylcholine diacyl C42:1 | 0.72 | 100.00 | 14.8% | Used | 0.32 ± 0.08 | 0.29 ± 0.08 | -0.17 | -0.20 |
| PC aa C42:2 | Phosphatidylcholine diacyl C42:2 | 0.56 | 100.00 | 14.6% | Used | 0.22 ± 0.06 | 0.21 ± 0.06 | -0.14 | -0.12 |
| PC aa C42:4 | Phosphatidylcholine diacyl C42:4 | 0.51 | 100.00 | 11.7% | Used | 0.22 ± 0.04 | 0.21 ± 0.04 | -0.09 | 0.01 |
| PC aa C42:5 | Phosphatidylcholine diacyl C42:5 | 0.75 | 100.00 | 10.6% | Used | 0.43 ± 0.11 | 0.41 ± 0.12 | -0.06 | 0.12 |
| PC aa C42:6 | Phosphatidylcholine diacyl C42:6 | 0.62 | 60.16 | 12.5% | Used | 0.64 ± 0.13 | 0.61 ± 0.13 | -0.12 | 0.04 |
| PC ae C30:0 | Phosphatidylcholine acyl-alkyl C30:0 | 0.76 | 98.86 | 18.1% | Used | 0.51 ± 0.14 | 0.44 ± 0.13 | -0.05 | -0.09 |
| PC ae C30:1 | Phosphatidylcholine acyl-alkyl C30:1 | 0.18 | 94.12 | 41.7% | Excluded |  |  |  |  |
| PC ae C30:2 | Phosphatidylcholine acyl-alkyl C30:2 | 0.65 | 86.34 | 17.5% | Used | 0.17 ± 0.04 | 0.14 ± 0.04 | 0.10 | 0.04 |
| PC ae C32:1 | Phosphatidylcholine acyl-alkyl C32:1 | 0.83 | 100.00 | 8.0% | Used | 3.02 ± 0.58 | 2.71 ± 0.53 | -0.12 | -0.06 |
| PC ae C32:2 | Phosphatidylcholine acyl-alkyl C32:2 | 0.77 | 100.00 | 11.6% | Used | 0.81 ± 0.18 | 0.68 ± 0.14 | -0.01 | 0.00 |
| PC ae C34:0 | Phosphatidylcholine acyl-alkyl C34:0 | 0.82 | 100.00 | 7.9% | Used | 1.82 ± 0.44 | 1.63 ± 0.38 | -0.02 | -0.09 |
| PC ae C34:1 | Phosphatidylcholine acyl-alkyl C34:1 | 0.87 | 100.00 | 7.6% | Used | 11.29 ± 2.19 | 9.82 ± 1.91 | -0.11 | -0.10 |
| PC ae C34:2 | Phosphatidylcholine acyl-alkyl C34:2 | 0.90 | 100.00 | 7.6% | Used | 13.71 ± 3.11 | 12.06 ± 2.99 | -0.14 | -0.14 |
| PC ae C34:3 | Phosphatidylcholine acyl-alkyl C34:3 | 0.91 | 100.00 | 7.9% | Used | 9.14 ± 2.42 | 8.05 ± 2.2 | -0.23 | -0.19 |
| PC ae C36:0 | Phosphatidylcholine acyl-alkyl C36:0 | 0.35 | 100.00 | 35.6% | Excluded |  |  |  |  |
| PC ae C36:1 | Phosphatidylcholine acyl-alkyl C36:1 | 0.85 | 100.00 | 9.8% | Used | 8.96 ± 2.06 | 7.87 ± 1.67 | -0.01 | -0.09 |
| PC ae C36:2 | Phosphatidylcholine acyl-alkyl C36:2 | 0.92 | 100.00 | 8.3% | Used | 16.64 ± 3.64 | 14.14 ± 3.36 | -0.17 | -0.21 |
| PC ae C36:3 | Phosphatidylcholine acyl-alkyl C36:3 | 0.86 | 100.00 | 8.1% | Used | 9.12 ± 1.95 | 8.28 ± 1.9 | -0.12 | -0.09 |
| PC ae C36:4 | Phosphatidylcholine acyl-alkyl C36:4 | 0.87 | 100.00 | 7.9% | Used | 20.54 ± 4.89 | 21.22 ± 5.47 | 0.04 | 0.05 |
| PC ae C36:5 | Phosphatidylcholine acyl-alkyl C36:5 | 0.89 | 100.00 | 8.0% | Used | 13.68 ± 3.42 | 14.03 ± 3.49 | 0.03 | 0.02 |
| PC ae C38:0 | Phosphatidylcholine acyl-alkyl C38:0 | 0.81 | 100.00 | 10.8% | Used | 2.6 ± 0.72 | 2.36 ± 0.7 | -0.04 | -0.10 |
| PC ae C38:1 | Phosphatidylcholine acyl-alkyl C38:1 | 0.48 | 100.00 | 12.4% | Used | 0.84 ± 0.27 | 0.79 ± 0.24 | -0.03 | -0.03 |
| PC ae C38:2 | Phosphatidylcholine acyl-alkyl C38:2 | 0.73 | 100.00 | 10.3% | Used | 2.3 ± 0.48 | 2.03 ± 0.44 | -0.11 | -0.14 |
| PC ae C38:3 | Phosphatidylcholine acyl-alkyl C38:3 | 0.85 | 100.00 | 9.2% | Used | 4.66 ± 0.99 | 4.03 ± 0.84 | 0.06 | -0.02 |
| PC ae C38:4 | Phosphatidylcholine acyl-alkyl C38:4 | 0.82 | 100.00 | 8.6% | Used | 16.18 ± 3.08 | 15.29 ± 2.99 | -0.04 | -0.06 |
| PC ae C38:5 | Phosphatidylcholine acyl-alkyl C38:5 | 0.82 | 100.00 | 8.3% | Used | 19.75 ± 4.04 | 20.04 ± 4.26 | -0.01 | -0.01 |
| PC ae C38:6 | Phosphatidylcholine acyl-alkyl C38:6 | 0.85 | 100.00 | 8.1% | Used | 8.82 ± 2.09 | 8.57 ± 2.05 | 0.04 | -0.03 |
| PC ae C40:0 | Phosphatidylcholine acyl-alkyl C40:0 | 0.87 | 1.05 | 4.8% | Used | 10.45 ± 1.48 | 9.99 ± 1.46 | -0.06 | -0.07 |
| PC ae C40:1 | Phosphatidylcholine acyl-alkyl C40:1 | 0.68 | 100.00 | 10.5% | Used | 1.7 ± 0.37 | 1.68 ± 0.39 | -0.12 | -0.14 |
| PC ae C40:2 | Phosphatidylcholine acyl-alkyl C40:2 | 0.85 | 100.00 | 9.5% | Used | 2.21 ± 0.51 | 2 ± 0.45 | 0.06 | -0.05 |
| PC ae C40:3 | Phosphatidylcholine acyl-alkyl C40:3 | 0.73 | 100.00 | 9.5% | Used | 1.24 ± 0.23 | 1.05 ± 0.21 | -0.07 | -0.12 |
| PC ae C40:4 | Phosphatidylcholine acyl-alkyl C40:4 | 0.82 | 100.00 | 9.6% | Used | 2.72 ± 0.51 | 2.5 ± 0.5 | -0.16 | -0.16 |
| PC ae C40:5 | Phosphatidylcholine acyl-alkyl C40:5 | 0.78 | 100.00 | 8.3% | Used | 3.69 ± 0.66 | 3.48 ± 0.65 | -0.16 | -0.19 |
| PC ae C40:6 | Phosphatidylcholine acyl-alkyl C40:6 | 0.88 | 100.00 | 8.6% | Used | 5.3 ± 1.23 | 4.86 ± 1.14 | -0.09 | -0.18 |
| PC ae C42:0 | Phosphatidylcholine acyl-alkyl C42:0 | 0.60 | 14.87 | 15.7% | Used | 0.52 ± 0.1 | 0.5 ± 0.1 | -0.07 | 0.02 |
| PC ae C42:1 | Phosphatidylcholine acyl-alkyl C42:1 | 0.51 | 100.00 | 11.5% | Used | 0.38 ± 0.09 | 0.37 ± 0.09 | -0.07 | 0.02 |
| PC ae C42:2 | Phosphatidylcholine acyl-alkyl C42:2 | 0.69 | 100.00 | 12.8% | Used | 0.7 ± 0.15 | 0.65 ± 0.14 | -0.15 | -0.14 |
| PC ae C42:3 | Phosphatidylcholine acyl-alkyl C42:3 | 0.80 | 100.00 | 10.8% | Used | 0.92 ± 0.19 | 0.84 ± 0.19 | -0.25 | -0.22 |
| PC ae C42:4 | Phosphatidylcholine acyl-alkyl C42:4 | 0.78 | 100.00 | 9.2% | Used | 1.08 ± 0.25 | 0.97 ± 0.25 | -0.25 | -0.23 |
| PC ae C42:5 | Phosphatidylcholine acyl-alkyl C42:5 | 0.86 | 99.97 | 7.4% | Used | 2.49 ± 0.5 | 2.29 ± 0.52 | -0.21 | -0.21 |
| PC ae C44:3 | Phosphatidylcholine acyl-alkyl C44:3 | 0.50 | 100.00 | 12.5% | Used | 0.12 ± 0.03 | 0.11 ± 0.03 | -0.17 | -0.12 |
| PC ae C44:4 | Phosphatidylcholine acyl-alkyl C44:4 | 0.71 | 100.00 | 11.4% | Used | 0.46 ± 0.11 | 0.41 ± 0.12 | -0.21 | -0.20 |
| PC ae C44:5 | Phosphatidylcholine acyl-alkyl C44:5 | 0.86 | 100.00 | 8.0% | Used | 2.21 ± 0.56 | 2.09 ± 0.6 | -0.18 | -0.19 |
| PC ae C44:6 | Phosphatidylcholine acyl-alkyl C44:6 | 0.89 | 100.00 | 7.7% | Used | 1.45 ± 0.38 | 1.34 ± 0.38 | -0.19 | -0.23 |
| lysoPC a C14:0 | lysoPhosphatidylcholine acyl C14:0 | 0.45 | 21.24 | 23.8% | Excluded |  |  |  |  |
| lysoPC a C16:0 | lysoPhosphatidylcholine acyl C16:0 | 0.75 | 100.00 | 8.8% | Used | 89.99 ± 18.13 | 99.79 ± 19.28 | -0.11 | -0.11 |
| lysoPC a C16:1 | lysoPhosphatidylcholine acyl C16:1 | 0.84 | 100.00 | 8.6% | Used | 2.83 ± 0.86 | 2.96 ± 1.08 | -0.05 | 0.03 |
| lysoPC a C17:0 | lysoPhosphatidylcholine acyl C17:0 | 0.84 | 100.00 | 12.7% | Used | 1.77 ± 0.49 | 1.74 ± 0.49 | -0.17 | -0.31 |
| lysoPC a C18:0 | lysoPhosphatidylcholine acyl C18:0 | 0.80 | 100.00 | 9.7% | Used | 25.35 ± 6.1 | 27.23 ± 5.68 | -0.04 | -0.14 |
| lysoPC a C18:1 | lysoPhosphatidylcholine acyl C18:1 | 0.84 | 100.00 | 9.2% | Used | 18.3 ± 5.05 | 21 ± 5.91 | -0.31 | -0.31 |
| lysoPC a C18:2 | lysoPhosphatidylcholine acyl C18:2 | 0.93 | 100.00 | 8.8% | Used | 25.86 ± 8.33 | 30.93 ± 10.18 | -0.34 | -0.37 |
| lysoPC a C20:3 | lysoPhosphatidylcholine acyl C20:3 | 0.77 | 100.00 | 9.0% | Used | 2.22 ± 0.61 | 2.6 ± 0.71 | -0.03 | -0.14 |
| lysoPC a C20:4 | lysoPhosphatidylcholine acyl C20:4 | 0.87 | 100.00 | 9.0% | Used | 6.16 ± 1.8 | 7.53 ± 2.27 | -0.11 | -0.17 |
| lysoPC a C24:0 | lysoPhosphatidylcholine acyl C24:0 | 0.09 | 12.45 | 21.1% | Excluded |  |  |  |  |
| lysoPC a C26:0 | lysoPhosphatidylcholine acyl C26:0 | 0.09 | 59.58 | 31.0% | Excluded |  |  |  |  |
| lysoPC a C26:1 | lysoPhosphatidylcholine acyl C26:1 | -0.04 | 0.00 | 7.9% | Excluded |  |  |  |  |
| lysoPC a C28:0 | lysoPhosphatidylcholine acyl C28:0 | 0.17 | 49.61 | 29.1% | Excluded |  |  |  |  |
| lysoPC a C28:1 | lysoPhosphatidylcholine acyl C28:1 | 0.29 | 99.84 | 22.6% | Excluded |  |  |  |  |
| lysoPC a C6:0 | lysoPhosphatidylcholine acyl C6:0 | -0.14 | 33.33 | 62.5% | Excluded |  |  |  |  |
| SM (OH) C14:1 | Hydroxysphingomyeline C14:1 | 0.91 | 100.00 | 7.7% | Used | 6.72 ± 1.6 | 5.61 ± 1.37 | 0.04 | -0.14 |
| SM (OH) C16:1 | Hydroxysphingomyeline C16:1 | 0.86 | 100.00 | 8.8% | Used | 3.63 ± 0.85 | 3.05 ± 0.7 | 0.07 | -0.10 |
| SM (OH) C22:1 | Hydroxysphingomyeline C22:1 | 0.82 | 100.00 | 11.2% | Used | 14.25 ± 2.95 | 12.55 ± 2.65 | 0.09 | -0.07 |
| SM (OH) C22:2 | Hydroxysphingomyeline C22:2 | 0.87 | 100.00 | 10.3% | Used | 12.68 ± 2.68 | 10.17 ± 2.2 | 0.06 | -0.13 |
| SM (OH) C24:1 | Hydroxysphingomyeline C24:1 | 0.75 | 100.00 | 15.1% | Used | 1.4 ± 0.34 | 1.28 ± 0.3 | 0.09 | -0.05 |
| SM C16:0 | Sphingomyeline C16:0 | 0.73 | 100.00 | 8.0% | Used | 108.87 ± 16.07 | 101.33 ± 16 | 0.00 | -0.11 |
| SM C16:1 | Sphingomyeline C16:1 | 0.84 | 100.00 | 7.5% | Used | 16.88 ± 2.99 | 14.54 ± 2.47 | 0.30 | 0.09 |
| SM C18:0 | Sphingomyeline C18:0 | 0.79 | 100.00 | 9.0% | Used | 23.98 ± 4.68 | 21.73 ± 4.23 | 0.20 | 0.14 |
| SM C18:1 | Sphingomyeline C18:1 | 0.84 | 100.00 | 8.2% | Used | 12.01 ± 2.56 | 10.06 ± 2.08 | 0.31 | 0.14 |
| SM C20:2 | Sphingomyeline C20:2 | 0.61 | 99.93 | 12.6% | Used | 0.43 ± 0.11 | 0.33 ± 0.09 | 0.13 | -0.04 |
| SM C22:3 | Sphingomyeline C22:3 | -0.04 | 55.85 | 57.6% | Excluded |  |  |  |  |
| SM C24:0 | Sphingomyeline C24:0 | 0.78 | 100.00 | 10.7% | Used | 21.58 ± 4.13 | 21.56 ± 4.24 | 0.08 | 0.04 |
| SM C24:1 | Sphingomyeline C24:1 | 0.75 | 100.00 | 10.0% | Used | 52.47 ± 9.36 | 51 ± 9.42 | 0.07 | 0.01 |
| SM C26:0 | Sphingomyeline C26:0 | 0.46 | 100.00 | 67.8% | Excluded |  |  |  |  |
| SM C26:1 | Sphingomyeline C26:1 | 0.69 | 100.00 | 20.8% | Used | 0.41 ± 0.13 | 0.41 ± 0.12 | 0.08 | 0.04 |
| H1 | Hexose | 0.69 | 100.00 | 6.3% | Used | 4809.34 ± 594.43 | 5100.89 ± 599.63 | 0.19 | 0.19 |

**Table S2. Potential biomarkers for age in men from KORA F4**

|  | **KORA F4 Females** | | |
| --- | --- | --- | --- |
| **marker** | **Mean ^1^ ± SD** | **beta^2^ (SE)** | **p-value** |
| C10:1 | 0.17±0.06 | 53.66 (6.12) | 7.62E-18 |
| C12:1 | 0.15±0.05 | 73.1 (6.97) | 1.63E-24 |
| C16:1 | 0.04±0.01 | 389.31 (35.98) | 6.26E-26 |
| C18:1 | 0.13±0.04 | 92.61 (10.15) | 3.67E-19 |
| Gln | 636.48±94.87 | -0.03 (0) | 5.87E-13 |
| His | 100.83±14.18 | -0.29 (0.03) | 6.15E-26 |
| Trp | 85.76±10.23 | -0.46 (0.04) | 3.67E-33 |
| Val | 293.31±51.56 | -0.09 (0.01) | 8.48E-31 |
| lysoPC a C18:2 | 30.93±10.18 | -0.36 (0.04) | 3.69E-18 |
| lysoPC a C20:3 | 2.6±0.71 | -5.07 (0.55) | 1.36E-19 |
| PC ae C36:1 | 7.87±1.67 | 1.53 (0.24) | 1.56E-10 |
| PC ae C42:0 | 0.5±0.1 | 29.29 (4.05) | 9.05E-13 |

^1^ mean concentration in µM from serum;

^2^ ß estimate represents changes per year of age, adjusted for BMI
